# Influence of GSH synthesis inhibition on temporal distribution of NAD+/NADH during vascular endothelial cells proliferation

**Published:** 2014

**Authors:** C Busu, V Atanasiu, G Caldito, TY Aw

**Affiliations:** * ”Carol Davila” University of Medicine and Pharmacy, Medical School, Biochemistry Department, Bucharest, Romania; ** Department of Molecular & Cellular Physiology, Louisiana State University Health Sciences Center, Shreveport, LA, USA

**Keywords:** GSH synthesis inhibition, NAD+/NADH, vascular endothelial cells, proliferation

## Abstract

Pathological conditions states such as stroke, diabetes mellitus, hypertension, dyslipidemia are associated with increased levels of free radicals that alter normal function of the vascular endothelium and perturb vascular homeostasis. The redox couples reduced glutathione (GSH)/oxidized glutathione (GSSG), NADH/NAD+, and NADPH/NADP+ play major functions in the intracellular redox balance. Any decrease in tissue or systemic GSH levels under the aforementioned pathologies would enhance oxidative damage to the vascular endothelium. Beside their role as coenzyme that participate in cellular metabolism, pyridine nucleotides serve also as substrate for enzymes involved in DNA repair and longevity. There is scant data on NAD+/NADH kinetics and distribution during human cells proliferation. Here, we determined the influence of cellular GSH status on the early dynamics of nuclear-to-cytosol (N-to-C) NAD+ and nuclear NADH kinetics (6h interval) over 72h of endothelial cell proliferation. The IHEC cell line was used as a surrogate for human brain micro vascular endothelial cells. Inhibition of GSH synthesis by buthionine sulfoximine (BSO) and sustained low cellular GSH significantly increased nuclear NADH levels (p<0.01), which correlated with lower nuclear GSH and prolonged cell cycle S-phase. When BSO was removed the pattern of nuclear NAD+ resembled that of control group, but nuclear NADH concentrations remained elevated, as in GSH deficient cells (p<0.01). The coincidence of high nuclear NADH and lower nuclear NAD+ with S-phase prolongation are suggestive of CtBP and NAD+-dependent DNA repair enzyme activation under conditions of decreased cellular GSH. These results provide important insights into GSH control of vascular endothelial growth and restitution, key processes in the restoration of the endothelium adjacent to the post-injury lesion site.

## Introduction

Vascular endothelial cells of the blood-brain barrier (BBB) are located at the interface between the vascular lumen and underlying tissues, and as such, are in direct contact with the systemic circulation. Hypoxia and lack or delayed glucose flux during stroke or induction of carbonyl stress in diabetes lead to increased vascular permeability and cerebral edema, elevated levels of systemic or locally generated mediators, free radicals, and reactive oxygen or carbonyl species [**1**]; consequently, these pathologies are among the leading causes of long-term disability and death in Western society. Therefore, the preservation or post-injury restoration of endothelial barrier integrity is central to the maintenance of endothelial function.

Intracellular distribution of glutathione (GSH) between the cytosol and the nucleus is a crucial factor in cell growth and proliferation. Through S-glutathiolation, GSH affords an elegant mechanism that modulates the oxidative modification of redox active cysteines within proteins that regulates physiological processes [**2**]. Studies on cell cycle progression have documented that the intracellular redox environment varies from a more oxidized state at G0/early G1 to a reduced state as cells transition through S to G2/M phases [**3**,**4**]. In a previous paper we showed that inhibition of GSH synthesis in an IHEC cell line lengthened the resident time of endothelial cells in the S-phase of the cell cycle that was coincident with decreased nuclear GSH, higher localization of cdk1 in the cytosol, as well as activation of nuclear chk2 and increased nuclear-to-cytosol GAPDH distribution, factors that are involved in DNA damage response and repair [**5**].

Redox homeostasis of a cell is maintained not only by the thiol redox couple glutathione/glutathione disulfide (GSH/GSSG), but also by redox couples like reduced and oxidized thioredoxin (Trx/TrxSS) and pyridine nucleotides (NAD+/NADH, NADP+/NADPH) [**6**]. Pyridine nucleotides are characterized mainly as electrons carriers in oxidoreductase reactions, but they are also important in cellular signaling and defense systems. NAD+ functions as a substrate for: 1) ADP-ribose transferases (ARTs) or poly (ADP-ribose) polymerases (PARPs), 2) cADP-ribose synthases, and 3) sirtuins (histone deacetylases) [**7**]. Through interacting with these enzymes, NAD+ and NADH play an important role in calcium homeostasis, DNA repair and gene expression [**8**].

Given that the NADH/NAD+ ratio reflects the cellular metabolic status, the redox state and NAD+ and NADH levels are therefore important in cell proliferation [**9**]. In the current study, we addressed the question how cellular GSH depletion affects the kinetics of cytosol– to-nuclear distribution of NAD+ and the nuclear concentration of NADH. We used a human brain endothelial cell (IHEC) line as surrogate of the blood-brain barrier and selected 6h time intervals post-seeding for all determinations throughout a 72h culture. In our study, the selection of the 6h time interval is significant and contrasts with earlier studies looking only 24h time intervals [**10**,**11**], which miss the early kinetics and dynamics of redox (GSH, pyridine nucleotides) fluxes. At present, there is scant information on NAD+ and NADH distribution between the cytoplasm and the nucleus under conditions of normal, decreased and restored GSH levels during the growth cycle of IHEC cells. Our hypothesis, based on our previous published results (5) was that during GSH deprivation endothelial cells adopt a mechanism that involves pyridine nucleotide nuclear distribution that could allow cells to preserve a reducing nuclear environment to protect their genome and survive. The current results show that in GSH depleted states an increased of nuclear NADH and nuclear NADH/NAD+ ratio occurred that correlated with a slowing of cell cycle progression evidenced by the lengthening of the S-phase.

## Methods

Reagents

Medium 199, L-buthionine-(S,R)-sulfoximine (BSO), insulin-transferrin-sodium selenite solution, propidium iodine (PI), iodoacetic acid (IAA), 2,4-dinitrophenyl fluorobenzene (DNFB), ethanol, trichloroacetic acid (TCA), imidazole, sucrose, RNase A, NAD+, NADH, ammonium acetate, bathophenanthroline-sulphonic acid, KCN, methanol were purchased from Sigma-Aldrich (St. Louis, MO). Digitonin was purchased from Wako, Japan. Fetal bovine serum (FBS) was obtained from Atlanta Biologicals (Lawrencevill, GA). Trypsin-EDTA and antibiotic/antimycotic were from Gibco. The Bio-Rad Protein Assay kit was from Bio-Rad Laboratories (Hercules, CA). Amicon Ultra filters were from Millipore Corporation.

Cell culture conditions

The immortalized human brain microvascular endothelial cell line (IHEC) was obtained from Dr. Danica Stanimirovic of the National Research Council of Canada's Institute for Biological Sciences and was propagated by Dr. Steven J. Alexander at LSUHSC. IHECs were maintained in 75 cm2 culture flasks in M-199 supplemented with 10% FBS, 1% insulin-transferrin-sodium selenite solution, and 1X antibiotic/antimycotic. Cells were incubated in humidified atmosphere with 5% CO2 at 370C. Media was changed every 2 days

Cell incubation for experiments

For all experiments, cells from confluent IHEC monolayers were seeded at a density of 2X105/well in 6- well plates up to 72 hours. Cells were collected at designated time points as follows: 0 (seeding moment), 30, 36, 42, 48, 55, 60, 66, 72 hours after seeding. To inhibit GSH synthesis, cells were treated with 10µM BSO, final concentration, at 4 and 24h after seeding (Treated group). Sustained depletion of GSH was maintained with the addition of 2µM BSO to the fresh media at 28 hours. Post-BSO treatment, a change to fresh media at 28 hours without additional BSO represented the Reversal group. The control group was without BSO treatment but with a change to fresh media at 28h post seeding.

Cell cycle analysis by flow cytometry

At the end of each time point, cells were trypsinized, washed with PBS and fixed in 70% cold ethanol. Fixed cells were spun at 40C, 1200 rpm for 10 minutes to remove the ethanol and resuspended in 1 ml PBS, after they were incubated in 1mL, 1mg/mL DNAase-free RNAase for 40 minutes in 370C water bath. 1ml propidium iodine (PI, 50µg/ml) was added and samples were incubated at 40C for at least 15 minutes in the dark. Flow cytometry analysis was performed by using a FACS Vantage flow cytometer (Becton Dickinson, San Jose, CA). A minimum of 1.5x106 cells per sample was counted. Data were processed by using Cell Quest software and gated on pulse-processed PI signals to exclude doublets and large aggregates, by using a multiparameter gate strategy.

Cellular fractionation

Separation of cytosolic and nuclear fractions was achieved by digitonin fractionation in 1.5 ml eppendorf tubes, using the method of Bronfan et al [**12**]. Cells were trypsinized and counted. Cells (3x106) were resuspended in 0.5 mL of fractionation buffer A, containing 0.25M sucrose, 3mM imidazole, pH=7.4. Digitonin (1mg/ml) was added to buffer A at this step to selectively permeabilize the plasma membrane. When cell samples were used for GSH measurements, 8mM IAA final concentration was included in the trypsinization step and in buffer A to prevent non-specific oxidation of GSH during workup. Cells were centrifuged at 14,000g, 40C (20s) and the supernatants represented the cytosolic fraction. In all instances following digitonin permeabilization, cell pellets were resuspended in 0.5 mL buffer A and homogenized with a Dounce homogenizer (5-10 passes The pellets were resuspended in 0.5mL buffer A and centrifuged and collected twice (14,000g, 40C, 2s). The final nuclear pellets were then washed twice with 0.5mL PBS and collected by centrifugation (14,000g, 40C, 2s). For GSH measurements nuclear pellets and cytosolic fractions were treated with 5% cold TCA, final concentration. The acid soluble supernatants were used for GSH determination by HPLC (see below).

For pyridine nucleotides determinations nuclear pellets were resuspended in buffer A containing 0.2 M KCN buffer (0.2M KCN, 0.06M KOH, 0.001M bathophenanthroline-sulphonic acid, H2O) to stabilize the pyridine nucleotides as nicotinamide-cyanide derivatives. The KCN buffer was added directly to cytosolic fractions. The purity of the nuclear and cytosolic extracts was validated by enrichment of H1 on western blots and by LDH assay, respectively.

GSH quantification

Cellular GSH level was determined by high-performance liquid chromatography (HPLC), using the method of Reed et al [**13**]. The TCA soluble cell extracts were derivatized with 6mM IAA and 1% 2, 4 DNFB to yield the S-carboxymethyl and 2, 4-dinitrophenyl derivative of GSH, respectively. Separation of GSH derivatives was performed on a 250x4.6mm Alltech Lichrosorb NH2 10-µm anion-exchange column. GSH contents were quantified by comparison to standards derivatized in the same manner. Protein pellets were resuspended in 1 mL 0.1M NaOH for protein quantification, and GSH levels are expressed as nmol/mg protein.

Quantification of NAD+, NADH

Cellular NAD+, NADH level were determined by HPLC, using the method of Unemura et al [**14**]. To nuclear or cytosolic extracts were added 200ul KCN buffer and mixture placed on ice for 5 minutes. Lipids were removed by chloroform extraction. After centrifugation (14000g, 40C, 5 min), the lipid-free upper layer was collected, transferred to Amicon Ultra filters, and the filtrate recovered by centrifugation (14,000g, 40C, 35 min). The cytosolic or nuclear filtrates (100μl) were mixed with 100 μl mobile phase (0.2 M ammonium acetate, MeOH; pH 6.0) and NAD+ and NADH were separated on a reversed- phase C18 column (250x4.6mm) and detected at 328nm (Gilson 118 UV=Vis detector). Pyridine nucleotide levels were quantified by comparison to standards and expressed asnmol/mg protein.

Protein assay

Proteins were measured by using the Bio-Rad Protein Assay kit (Bio-Rad Laboratories, Hercules, CA), according to the manufacturer’s protocol. GSH and NAD+, NADH were expressed as nmoles/mg protein.

Statistical analysis

Two-Factor Analysis of Variance (ANOVA) was used to determine significance among groups namely, Control, GSH depleted (Treated) and GSH restored (Reversal), times (30, 36, 42, 48, 55, 60, 66 and 72 h), and interaction between group and time. For each of the three variables, multiple comparisons among the 3 groups and among the 8 time points were performed using the Bonferroni test. Results are expressed as mean ± SEM. The relationships between nuclear GSH, NAD+ and NADH were analyzed by Pearson Correlation.

## Results

The influence of cellular GSH manipulation on cytosolic and nuclear NAD+ concentrations and cell cycle S phase

Nuclear and cytosolic NAD+ contents and also nuclear-to-cytosol NAD+ ratio were determined in parallel with GSH concentration and cell cycle S phase. Effects of group and group time interaction are presented in **Tables 1**-**3**.

**Table 1:** Effects of Group and Group-Time Interaction among control cell, GSH depleted (Treated) cells and GSH restored (Reversal) cells for **nuclear NAD+**

**Effect**	**N 1**	**Mean ± SD 2 **	**p-value**
Group			<0.01**
WT Control	40	0.231 ± 0.050	
Reversal	40	0.229 ± 0.045	
BSO Treated	40	0.203 ± 0.064	
Time-Group Interaction			<0.01**
30 hour - Wt control	5	0.282 ± 0.012	
Reversal	5	0.383 ± 0.029 a	
BSO Treated	5	0.313 ± 0.073	
36 hour - Wt control	5	0.235 ± 0.024	
Reversal	5	0.281 ± 0.026	
BSO Treated	5	0.252 ± 0.018	
42 h - Wt control	5	0.259 ± 0.053	
Reversal	5	0.247 ± 0.037	
BSO Treated	5	0.252 ± 0.018	
48 h - Wt control	5	0.249 ± 0.044	
Reversal	5	0.258 ± 0.023	
BSO Treated	5	0.209 ± 0.037	
55 h - Wt control	5	0.258 ± 0.034	
Reversal	5	0.243 ± 0.025	
BSO Treated	5	0.175 ± 0.029 b	
60 h - Wt control	5	0.227 ± 0.034	
Reversal	5	0.214 ± 0.013	
BSO Treated	5	0.167 ± 0.026 b	
66 h - Wt control	5	0.195 ± 0.010	
Reversal	5	0.207 ± 0.031	
BSO Treated	5	0.150 ± 0.025 b	
72 h - Wt control	5	0.146 ± 0.015	
Reversal	5	0.230 ± 0.029 c	
BSO Treated	5	0.148 ± 0.029	
1- N- number of observations			
2 - SD- standard deviation			
a – reversal significantly higher than control and treated groups			
b – treated significantly lower than control and reversal groups			
c - reversal significantly higher than control and treated groups			

**Table 2:** Effects of Group and Group-Time Interaction among control cell, GSH depleted (Treated) cells and GSH restored (Reversal) cells for **cytosolic NAD+**

**Effect**	**N 1**	**Mean ± SD 2 **	**p-value**
Group			0.11 NS
WT Control	40	2.010 ± 0.972	
Reversal	40	2.232 ± 0.970	
BSO Treated	40	2.030 ± 0.743	
Time-Group Interaction			<0.01**
30 hour - Wt control	5	2.974 ± 0.617	
Reversal	5	3.577 ± 0.337 a	
BSO Treated	5	2.408 ± 0.652	
36 hour - Wt control	5	3.463 ± 1.617	
Reversal	5	3.830 ± 0.551	
BSO Treated	5	2.540 ± 0.710	
42 h - Wt control	5	2.199 ± 0.053	
Reversal	5	3.229 ± 0.683	
BSO Treated	5	2.677 ± 0.620	
48 h - Wt control	5	1.893 ± 0.158	
Reversal	5	2.835 ± 0.550	
BSO Treated	5	2.634 ± 0.499	
55 h - Wt control	5	1.595 ± 0.189	
Reversal	5	2.061 ± 0.199	
BSO Treated	5	2.017 ± 0.432	
60 h - Wt control	5	2.017 ± 0.029	
Reversal	5	1.593 ± 0.138	
BSO Treated	5	1.540 ± 0.208	
66 h - Wt control	5	1.176 ± 0.292	
Reversal	5	1.524 ± 0.157	
BSO Treated	5	1.202 ± 0.129	
72 h - Wt control	5	1.202 ± 0.058	
Reversal	5	1.568 ± 0.084	
BSO Treated	5	1.221 ± 0.337	
1- N- number of observations			
2 - SD- standard deviation			
a – reversal significantly higher than control and treated groups			

**Table 3:** Effects of Group and Group-Time Interaction among control cell, GSH depleted (Treated) cells and GSH restored (Reversal) cells for **ratio of nuclear to cytosol NAD+**

**Effect**	**N 1**	**Mean ± SD 2 **	**p-value**
Group			<0.01**
WT Control	40	0.1285 ± 0.0375	
Reversal	40	0.1136 ± 0.0305	
BSO Treated	40	0.1050 ± 0.0244	
Time-Group Interaction			<0.01**
30 hour - Wt control	5	0.0989 ± 0.0244	
Reversal	5	0.1236 ± 0.0145	
BSO Treated	5	0.1318 ± 0.0177	
36 hour - Wt control	5	0.077 ± 0.0304	
Reversal	5	0.0738 ± 0.0045	
BSO Treated	5	0.1048 ± 0.0265	
42 h - Wt control	5	0.1174 ± 0.0163 a	
Reversal	5	0.0792 ± 0.0200	
BSO Treated	5	0.0784 ± 0.0140	
48 h - Wt control	5	0.1322 ± 0.0254 a	
Reversal	5	0.0929 ± 0.0136	
BSO Treated	5	0.0799 ± 0.0088	
55 h - Wt control	5	0.1620 ± 0.0157 a	
Reversal	5	0.1195 ± 0.0205	
BSO Treated	5	0.0887 ± 0.0168	
60 h - Wt control	5	0.1452 ± 0.0288 a	
Reversal	5	0.1350 ± 0.0081	
BSO Treated	5	0.1086 ± 0.0076	
66 h - Wt control	5	0.1726 ± 0.0358	
Reversal	5	0.1374 ± 0.0268	
BSO Treated	5	0.1241 ± 0.0169	
72 h - Wt control	5	0.1216 ± 0.0133	
Reversal	5	0.1470 ± 0.0144	
BSO Treated	5	0.1236 ± 0.0145	
1- N- number of observations			
2 - SD- standard deviation			
a – control significantly higher than reversal and treated groups			

**Fig. 1:**
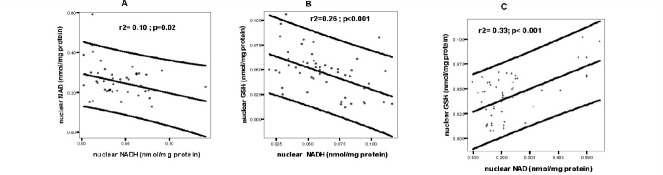
Linear regression line, Pearson correlation coefficient and p values of nuclear fractions from IHEC s, cells treated with BSO to maintain low GSH (Treated) or treated with BSO that was removed to allow GSH recovery (Reversal). These comparisons are the only that show significance. **A** - Correlations between nuclear NAD and NADH in control group; **B** - Correlations between nuclear GSH and NADH in reversal group; **C** - Correlations between nuclear GSH and NAD in treated group

In the BSO treated group, the mean value of nuclear NAD+ was significantly lower as compared to either the reversal or control group (p<0.01) (**Table 1**). In addition there was a significantly positive correlation between nuclear GSH and nuclear NAD+ (p<0.001) (**Fig. 1C**). The mean value of nuclear-to-cytosol NAD+ ratio for the BSO treated group was significantly lower than that for the reversal or control groups (p<0.01) (**Table 3**). At 42, 48, 55, 60 and 66 hours, the mean value for nuclear-to-cytosol NAD+ ratios in the control group were significantly higher than those for the reversal and treated groups (p<0.01) (**Table 3**).

**Fig. 2:**
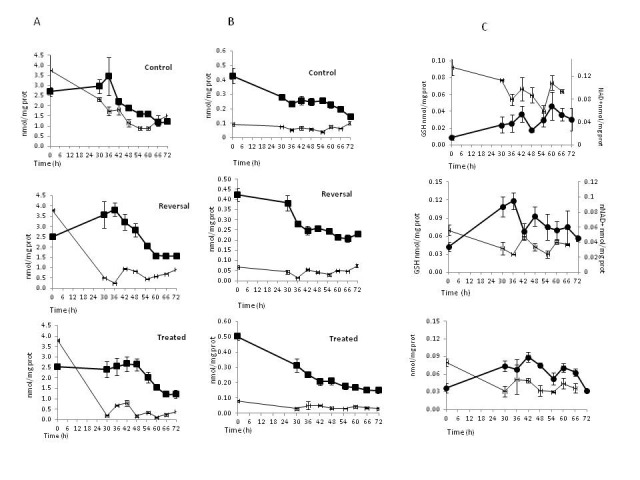
Time course of cytosolic (A) and nuclear (B, C) GSH and NAD+, NADH from 0 to 72 h in cytosolic and nuclear fractions from IHEC cells treated with BSO to maintain low GSH (Treated) or treated with BSO that was removed to allow GSH recovery (Reversal). Line drawing represents GSH data reproduced from ref. 5 and included here for direct comparison with changes in cytosolic & nuclear NAD+ and NADH. Filled square represents NAD+; filled circle represents NADH. Results are mean ± SEM for 6 (GSH) or 5 (NAD+, NADH) different determinations. Vertical error bars represent mean ± SEM of GSH, NAD+, NADH

**Fig. 3:**
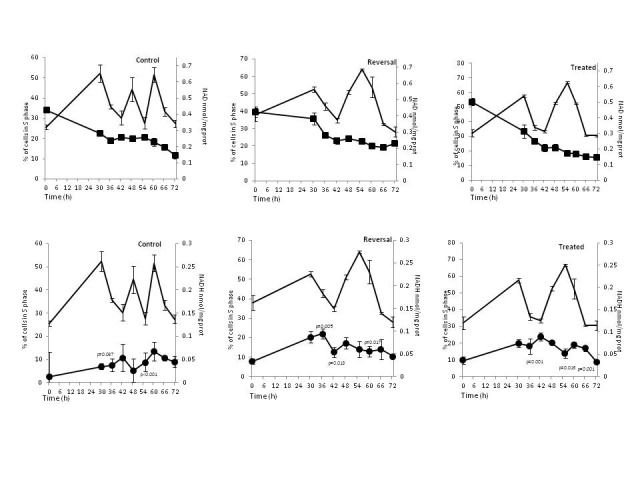
Temporal relationship between nuclear NAD+, NADH and S phase. Cells were treated with BSO to maintain low GSH (Treated) or treated with BSO that was removed at 28h to allow GSH recovery (Reversal). Line drawing represents S-phase reproduced from ref .5 and included here for direct comparison with changes in nuclear NAD+ and NADH. Filled square represents NAD+; filled circle represents NADH. Results are mean ± SEM for 5 (NAD+, NADH) or 6 (S phase) different determinations. Vertical error bars represent mean ± SEM of NAD+, NADH, % of cell in S phase. p is correlation coefficient between NADH and % of cell in S phase

The control group maintained a relatively constant concentration of nuclear NAD+ between 36-66h (**Fig. 2B**), a time frame that corresponded with two rounds of cell cycle as showed by S phase peaks (**Fig. 3A**). In the reversal group constant levels of nuclear NAD+ were maintained between 42-72h (**Fig. 2B**), which correlated with a lengthened S phase of the cell cycle (**Fig. 3B**)

There was a constant gradual decrease in nuclear NAD+ (**Fig. 2B**) that negatively correlated with GSH values (p<0.001; **Fig. 1**) in the BSO treated group.

Effects of cellular GSH depletion on nuclear NADH concentrations and cell cycle S phase

Effects of group and time - group interaction on nuclear NADH among control, treated and reversal groups are shown in **Table 4**.

**Table 4:** Effects of Group and Group-Time Interaction among control cell, GSH depleted (Treated) cells and GSH restored (Reversal) cells for **nuclear NADH**

**Effect**	**N 1**	**Mean ± SD 2 **	**p-value**
Group			<0.01**
WT Control	40	0.0451 ± 0.0274 a	
Reversal	40	0.0650 ± 0.0241	
BSO Treated	40	0.0655 ± 0.0218	
Time-Group Interaction			<0.01**
30 hour - Wt control	5	0.0349 ± 0.0241 b	
Reversal	5	0.0796 ± 0.0165	
BSO Treated	5	0.0736 ± 0.0161	
36 hour - Wt control	5	0.0364 ± 0.0274 b	
Reversal	5	0.0949 ± 0.0186	
BSO Treated	5	0.0682 ± 0.0300	
42 h - Wt control	5	0.0534 ± 0.0243	
Reversal	5	0.0544 ± 0.0207	
BSO Treated	5	0.0890 ± 0.0153	
48 h - Wt control	5	0.0257 ± 0.0056 b	
Reversal	5	0.0739 ± 0.0226	
BSO Treated	5	0.0751 ± 0.0072	
55 h - Wt control	5	0.0444 ± 0.0204	
Reversal	5	0.0595 ± 0.0300	
BSO Treated	5	0.0517 ± 0.0177	
60 h - Wt control	5	0.0677 ± 0.0440	
Reversal	5	0.0562 ± 0.0200	
BSO Treated	5	0.0711 ± 0.0119	
66 h - Wt control	5	0.0528 ± 0.0187	
Reversal	5	0.0562 ± 0.0200	
BSO Treated	5	0.0630 ± 0.0100	
72 h - Wt control	5	0.0451 ± 0.0338	
Reversal	5	0.0453 ± 0.0079	
BSO Treated	5	0.0322 ± 0.0076	
1N – number of observations			
2SD – Standard Deviation			
a – control group is significantly lower than other 2 groups			
b- control group is significantly lower than other 2 groups			

The mean of the control group was significantly lower (p<0.01) than those of reversal and treated groups, which were similar. Analysis of the time - group interactions revealed that at 30, 36 and 48 hours the mean values of the reversal and treated groups were significantly higher than that of control (p<0.01) (**Table 4**). The means of nuclear NADH- to- NAD+ ratio of control and reversal groups were similar and each was significantly lower than that for BSO treated (p<0.01) (**Table 5**). At 30, 36, 48 and 55 hours ratio mean values for control were significantly lower than those for reversal and treated groups which were similar, while at 42, 60 and 66 hours ratio values for treated group were significantly higher than those for the reversal and control groups (p<0.01) (**Table 5**).

**Table 5:** Effects of Group and Group-Time Interaction among control cell, GSH depleted (Treated) cells and GSH restored (Reversal) cells for **nuclear NADH to NAD+ Ratio**

**Effect**	**N 1**	**Mean ± SD 2 **	**p-value**
Group			<0.01**
WT Control	40	0.2062 ± 0.1440	
Reversal	40	0.2511 ± 0.0845	
BSO Treated	40	0.3378 ± 0.1145 a	
Time-Group Interaction			<0.01**
30 hour - Wt control	5	0.1249 ± 0.0864 b	
Reversal	5	0.1930 ± 0.0176	
BSO Treated	5	0.2456 ± 0.0745	
36 hour - Wt control	5	0.1603 ± 0.1340 b	
Reversal	5	0.3362 ± 0.0416	
BSO Treated	5	0.2673 ± 0.1051	
42 h - Wt control	5	0.2012 ± 0.0607	
Reversal	5	0.2195 ± 0.0641	
BSO Treated	5	0.4407 ± 0.0910 c	
48 h - Wt control	5	0.1075 ± 0.0370 b	
Reversal	5	0.2826 ± 0.0660	
BSO Treated	5	0.3658 ± 0.0583	
55 h - Wt control	5	0.1686 ± 0.0629 b	
Reversal	5	0.2538 ± 0.1433	
BSO Treated	5	0.2977 ± 0.0925	
60 h - Wt control	5	0.2976 ± 0.1971	
Reversal	5	0.2638 ± 0.0952	
BSO Treated	5	0.4267 ± 0.0467 c	
66 h - Wt control	5	0.2727 ± 0.1010	
Reversal	5	0.2638 ± 0.0952	
BSO Treated	5	0.4346 ± 0.1191 c	
72 h - Wt control	5	0.3168 ± 0.2499	
Reversal	5	0.1959 ± 0.0225	
BSO Treated	5	0.2243 ± 0.0630	
1N – number of observations			
2SD – Standard Deviation			
a –treated group significantly higher than other 2 groups			
b- control group is significantly lower than other 2 groups			
c – treated group is significantly higher than other 2 groups			

Nuclear GSH was higher in the control group and its peaks at 42 and 60h correlated with those for NADH (**Fig. 2C**). There was a negative correlation between nuclear NAD+ and nuclear NADH in the control group (p=0.02) (**Fig. 1**), but a positive correlation for NADH and S phase at 30h (Pearson correlation=0.838; p=0.037) and 55h (Pearson correlation=0.993; p<0.001) (**Fig. 3**). GSH depletion led to an increase in NADH and their patterns were similar to each other, with higher levels between 30-48 and 55-66 hours (**Fig. 2C**) and a positive correlation with S phase at 36h (Pearson correlation=0.980; p=0.001), 60h (Pearson correlation=0.905; p=0.013) and 72h (Pearson correlation=0.757; p=0.001) (**Fig. 3**). When GSH synthesis capacity was restored, the higher concentrations of NADH persisted and covered the time between 42 and 66 hours and negatively correlated with GSH values (p<0.001; **Fig. 1**); also there was a positive correlation between NADH and S phase of cell cycle at 36h (Pearson correlation=0.942; p=0.005), 42h (Pearson correlation=0.905; p=0.013) and 60h (Pearson correlation=0.892; p=0.017) (**Fig. 3**).

## Discussion

In this study, we have investigated the effect of cellular GSH status on cytoplasmic and nuclear distribution of NAD+ and nuclear levels of NADH and the relationship to S phase in the cell cycle. Our study is the first to demonstrate a link between GSH levels and intracellular distribution of pyridine nucleotides in brain endothelial cells. Most of the studies on redox couples and cell growth addressed this issue from a static view or at most convenient time points intervals, from 24 to 24hours, thus generating an incomplete image. Our study is a dynamic one, with multiple and close time points (6h time interval) which brings new insights into early stages of cellular proliferation in correlation with dynamic and kinetics of redox fluxes. Attenuation of GSH cellular synthetic capacity resulted in an increase in nuclear NADH pool size and a decrease in nuclear NAD+ one, with an augmentation of the nuclear NADH/NAD+ ratio. Besides its metabolic role, NAD+ also participates in signal transfer reactions as ADP-ribosyl transfers [**15**]. For instance, poly (ADP-ribose) polymerase (PARP) is a NAD+-cleaving enzyme that is important in DNA repair and genome stability [**16**,**17**], while Sirt1 is an NAD+ –dependent histone deacetylase that affects life span [**18**]. One explanation for the decrease in nuclear NAD+ pool that we have found in GSH insufficient cells might be its consumption in reaction catalyzed by NAD+-dependent enzymes as those aforementioned.

Carboxy-terminal binding protein (CtBP) is a transcriptional corepressor that may serve as a redox sensor through its ability to interact differently with NAD+ and NADH [**19**]. Thus, CtBP links transcription with cellular metabolism and its function it was shown to be augmented by high levels of nuclear NADH [**20**]. CtBP appears to be an important regulator of the E2F7-E2F1 regulatory loop that is activated by p53 during DNA damage and regulate cell proliferation and apoptosis by repression of cell cycle inhibitors and proapoptotic genes [**21**,**22**]. In our study, we found that under conditions of GSH depletion, nuclear NADH was significantly augmented and this might be explained by an increase requirement for CtBP activity.

Our results bring new insights into cellular GSH disruption and the redox environment of the nucleus. The current results support a suggestion that during GSH deficiency, endothelial cells increase their nuclear NADH levels in order to maintain a reduced redox state within the nucleus that would be necessary for DNA repair and cell survival. In a previous paper, we showed that a significant decrease in the total nuclear GSH content corresponded to the arrest of cells in the S-phase and activation of DNA damage response and repair as evidenced by elevated nuclear chk-2 phosphorylation (activation) and increased nuclear-to-cytosol GAPDH distribution, prior to peak cell arrest in S-phase [**5**]. By this new study, we have a better image of the impact of cellular GSH status on nuclear levels of redox couple NAD+/NADH with important roles in DNA repair, proliferation and survival.

The exactly mechanisms of the regulation of NADH increase and decreased NAD+ levels in GSH depleted cells are unclear and currently under investigation in our laboratory. Decreases in NAD+ have been found in ischemic brains [**23**] and intranasal NAD+ administration profoundly reduced infarct formation and neurological deficits [**24**]. Proliferation is a crucial step in endothelial layer repair after wounding and understanding how redox couples such as GSH and pyridine nucleotides influence one another will have key implications for oxidative stress-induced neurovascular pathologies like diabetes and neurodegenerative disease.

**Acknowledgment**

We thank Deborah Chervenak for technical assistance in flow cytometry. This study was supported by NIH Grant DK44510 (TYA).
